# FOXM1-regulated ZIC2 promotes the malignant phenotype of renal clear cell carcinoma by activating UBE2C/mTOR signaling pathway

**DOI:** 10.7150/ijbs.84067

**Published:** 2023-06-26

**Authors:** Zhengtong Lv, Miao Wang, Huimin Hou, Guyu Tang, Haozhe Xu, Xuan Wang, Yuan Li, Jianye Wang, Ming Liu

**Affiliations:** 1 Department of Urology, Beijing Hospital, National Center of Gerontology; Institute of Geriatric Medicine, Chinese Academy of Medical Sciences, P.R. China.; 2 Graduate School of Peking Union Medical College and Chinese Academy of Medical Sciences, P.R. China.; 3 Department of Urology, Xiangya Hospital, Central South University, Changsha, Hunan, P.R. China.; 4 Department of Urology, Second Xiangya Hospital, Central South University, Changsha, Hunan, P.R. China.

**Keywords:** Clear cell renal cell carcinoma, ZIC2, FOXM1, UBE2C, mTOR

## Abstract

**Background:** As a transcription factor, Zic family member 2 (ZIC2) has been involved in more and more studies of tumorigenesis, which has been proved by our research team to be an effective prognostic marker for Pan-cancer. However, the prognosis, tumor promoting effect and regulatory mechanism of ZIC2 in clear cell renal cell carcinoma (ccRCC) are still unknown.

**Methods:** The potential clinical significance of ZIC2 was evaluated by bioinformatics analysis using data from TCGA, GEO, and ArrayExpress data sets. WB and IHC were used to detect ZIC2 expression in tumors and adjacent tissues. CCK-8, EdU, colony formation, cell cycle, wound healing, transwell, subcutaneous xenograft, and lung metastasis models were used to detect the biological function of ZIC2. The regulatory mechanism of ZIC2 was confirmed by data of RNA-seq, ATAC-seq, MS-PCR, Chip-PCR, and luciferase reporter experiments.

**Results:** ZIC2 was markedly upregulated and correlated with poor clinicopathological features in ccRCC. Knockdown of ZIC2 resulted in reduced cell proliferation, invasion, migration, induction of G2/M phase arrest, and reduced tumor formation and lung metastasis in nude mice. The opposite was observed after overexpression. Mechanistically, the high expression of ZIC2 is regulated by hypomethylation and high H3K4Me3 in the promoter region, as well as positive transcriptional regulation by FOXM1. And then, ZIC2 transcriptase-positively regulates UBE2C and activates AKT/mTOR signaling pathway to promote tumor malignant progression.

**Conclusion:** This study reveals that FOXM1-ZIC2-UBE2C-mTOR signaling axis promotes the progression of ccRCC, which can be used as a prognostic indicator and potential therapeutic target.

## Introduction

Renal cancer is one of the top 10 causes of cancer-related deaths worldwide, resulting in more than 431,000 new cases and 179,000 deaths per year 1. Clear cell renal cell carcinoma (ccRCC), derived from the proximal convoluted renal tubules, is the most common subtype of renal cell carcinoma 2. The primary treatment for localized ccRCC is surgery, but approximately 30% of patients are found to have metastases during follow-up, and approximately 10% die of disease progression within 5 years after surgery 3. However, the incidence and mortality of metastatic and advanced ccRCC are high because they are not sensitive to traditional chemoradiotherapy. ccRCC is an immunogenic and angiogenic tumor. Immunotherapy and targeted therapy have changed the therapeutic prospect of advanced ccRCC 4, but the length of response and survival benefit of these therapies varies considerably among patients. Therefore, a better understanding of the underlying mechanisms in the pathogenesis of ccRCC could aid in the development of new therapeutic and diagnostic strategies.

Zic family member 2 (ZIC2), belonging to the Zic family of proteins, located on chromosome13q32.3. As a transcription factor, it interacts with a variety of DNA and proteins and was originally found to play an important role in human growth and development, especially in the central nervous system 5-8. In recent years, there are more and more reports on the involvement of ZIC2 in tumorigenesis. Recently, our research group has integrated 33 cancer species confirmed that ZIC2 is highly expressed in multiple tumors and had a significant correlation with tumor progression and survival of patients especially in renal cell carcinoma 9. To our best knowledge, the role of ZIC2 in ccRCC has not been fully described.

In the present study, we fully illustrate the prognostic value of ZIC2 expression in ccRCC through public datasets and local dataset. In addition, we fully explored the upstream regulatory mechanism of ZIC2 overexpression in ccRCC and its effect mechanism on downstream phenotype and found that ZIC2 overexpression originates from the opening of transcriptional regulatory region and the positive transcriptional regulation of FOXM1. Also, as a transcription factor, highly expressed ZIC2 further positively regulates the transcriptional expression of UBE2C and activates mTOR signaling pathway to promote the malignant phenotype of ccRCC. To sum up, FOXM1-ZIC2-UBE2C-mTOR axis has oncogenic activity and can be used as a potential therapeutic target and prognostic biomarker for ccRCC.

## Materials and methods

### Data acquisition

The gene expression profiles (HTSeq-FPKM data) and related clinical information of ccRCC were searched from TCGA Data Portal (http://cancergenome.nih.gov/), containing 531 ccRCC tissues and 72 matched adjacent normal renal tissues. In addition, we also selected several geo datasets to verify our finding, including GSE781, GSE6344, GSE14762, GSE17895, GSE36895, GSE40435, GSE46699, GSE53000, GSE53757, GSE66272, GSE68417, GSE71963, GSE73731, GSE89563, GSE105261, GSE126964, GSE150404. The datasets of E-MTAB-1980 10 from ArrayExpress was also used to validate the prognostic value. All the included database information is shown in Table S1. Twenty pairs of ccRCC tumor tissues and corresponding normal renal tissues were obtained from surgical resection specimens. The study was approved by the ethics committee of Beijing Hospital (2022BJYYEC-406-01).

### Cell culture and transfection

Four ccRCC cell lines ACHN, 786-O, Caki-1, and 769-P were used in this study. ACHN was cultured in Eagle's Minimum Essential Medium (EMEM). 786-O and 769-P cells were maintained in RPMI-1640 medium. Caki-1 was cultured in McCoy's 5A Medium. All media were supplemented with 10% fetal bovine serum and 1X antibiotics (penicillin and streptomycin). Cell lines were maintained in a humidified atmosphere at 37 °C and 5% CO2. Lentiviral vectors with ZIC2 and FOXM1 short hairpin RNA (shRNA) were purchased from Bainuo Biotechnological Co., Ltd (Zhengzhou, China). The target ZIC2 and FOXM1 shRNA sequences were shown in Table S2. Lentiviral infection referred to the manufacturer's protocol.

### Quantitative RT-PCR

RT-qPCR was performed as previously described 11. Total RNA was extracted according to the instructions for Trizol reagent (Invitrogen, Waltham, MA, United States). PrimeScript RT Master Mix [Takara Biotechnology (Dalian) Co., Ltd., Japan] were reverse transcribed into cDNA, and finally qRT-PCR assay was performed according to Premix Ex TaqTM II [Takara Biotechnology (Dalian) Co., Ltd.]. GAPDH was used as an internal reference gene to calibrate relative expression levels. The sequence of primers of genes to be detected is shown in Table S3.

### Western blotting

Protein samples (50 μg) were separated by sodium dodecyl sulfate-polyacrylamide gel electrophoresis in 4%-12% gel and transferred to nitrocellulose membranes for reaction with antibodies against target genes (Table S4). Following incubation with respective secondary antibodies (LI-COR Bioscences, Lincoln, NE), and scanned by Odyssey® CLx equipment (LI-COR Bioscences).

### Immunohistochemistry

Surgically resected specimens were first fixed in 10% formaldehyde, embedded in paraffin and routinely dehydrated. Paraffin sections were made into 4μm thick. Primary antibody ZIC2 was added dropfold to the tissue sections, followed by incubation and washing. Secondary antibody was added dropfold to the tissue sections, followed by incubation, washing, DAB color development, termination of reaction, hematoxylin counterstained, dehydration, transparency, sealing, microscope observation and photography. The score was based on the staining intensity of the positive cells: Negative (0 score), Weak (1 score), Moderate (2 score) and Strong (3 score) expression.

### Immunofluorescence

The transfected cells to be examined were fixed for 15 minutes at room temperature using 4% paraformaldehyde. Subsequently, the cells were washed with phosphate buffered saline (PBS) and permeabilized with 0.5% Triton X-100. To minimize non-specific antibody binding, the cells were blocked with 10% goat serum for 60 minutes at room temperature. The cells were then incubated overnight at 4 °C with a primary antibody specific to E-cadherin. Following a wash with PBS, the cells were incubated for 1 hour with an appropriate fluorochrome-conjugated secondary antibody. Nuclear staining was performed using DAPI, and the cells were observed under a fluorescence microscope.

### Cell proliferation evaluation

The CCK-8 and EdU assay were used to reflect cell proliferation. For CCK-8 analysis, cells were seeded in 96-well plates and 10 μL CCK-8 reagent was added to each well after 24, 48, 72, and 96 h, respectively. Measure the absorbance at 450 nm (OD value). The EdU assay was completed according to the manufacturer's recommendations (Ruibo, China).

### Colony formation assay

The logarithmic growth phase of ccRCC cells was seeded in a 12-well plate at a density of 500 cells per well, or in a 6-well plate at a density of 800 cells per well, and cultured at 37 °C. The culture continued for 1-3 weeks, during which the solution was changed every 3 days and the cell status was observed every day until the number of cells in most single clones was greater than 50. Then the cells were cleaned with phosphate-buffered saline buffer, made in methanol approximately 30 min, and stained with crystal violet dye at a dose of 1%. For clone counting, automated clone counting was performed using ImageJ 1.51d software.

### Cell cycle detection by Flow cytometry

The cells in logarithmic growth phase were washed three times with PBS and then the corresponding medium without serum FBS was added. Then the cells were fixed with precooled 75% ethanol and placed at 4 °C overnight. On the second day, ethanol was removed by centrifugation, and after washing with PBS, 100 μL RNase A was added and placed in a 37 °C water bath for 30 min. Then propidium iodide staining solution was added and the reaction was sealed in the refrigerator at 4 °C for 1 hour in the dark. Finally, the cell cycle distribution was determined according to the content of DNA in each stage of the cell.

### Wound healing assay

To conduct the wound healing assays, the cells were plated on 6-well plates and grew to 90% fusion. Scratch the cells with the head of a pipette gun, and PBS is added to wash and remove floating cells and cell debris. Cell images were obtained at 0h and 48h after wounding, and cell migration was observed.

### Transwell invasive assay

Cell invasion activity was assessed by transwell plates coated with extracellular interstitial gel. Logarithmic growth cells were cultured in serum-free medium in the transwell superior lumen, and a fresh medium containing 10% FBS was placed in the lower chamber as a chemical attractant. After 24h culture, the cells migrated to the lower membrane surface were fixed with 4% paraformaldehyde and stained with 0.1% crystal violet.

### Methylation-Specific PCR (MS-PCR)

To assess the methylation status of ZIC2, methylation-specific PCR (MS-PCR) was performed. Genomic DNA was collected from the tumor and adjacent tissues of 3 pairs of patients and was modified by bisulfite. Methylated-specific and unmethylated-specific primers were determined based on the position of cg16584172 (Table S3). A positive control of methylation was achieved by using the EpiTect MSP kit (Qiagen, USA). A negative control of methylation was achieved by using distilled water.

### Chromatin immunoprecipitation (ChIP) assay

SimpleChIP® Plus Enzymatic Chromatin IP Kit (Magnetic Beads) was used for chromatin immunoprecipitation. In brief, tumor cells in the logarithmic growth phase were taken and fixed with 1% formaldehyde for 10 min; then, cells were incubated with glycine for 5 min to terminate cross-linking. The medium was removed, and the cells were washed twice with PBS and collected by centrifugation after addition of PBS and protease inhibitors. The DNA was digested by Micrococcal Nuclease to a length of approximately 150 to 900 bp. Chromatin extracts were immunoprecipitated using anti-FOXM1 and anti-ZIC2 antibodies. Histone H3 (D2B12) XP® Rabbit mAb #4620 was used as a positive control and Normal Rabbit IgG #2729 as a negative control. Primers were designed for potential binding sites in the promoter regions of ZIC2 and UBE2C for PCR (Table S3).

### Luciferase reporter assay

The binding of FOXM1 to the ZIC2 promoter and the binding of ZIC2 to the UBE2C promoter were verified by Dual-luciferase reporter assay. The wild type and mutant promoter binding region sequences were constructed into the luciferase system. The reporter gene plasmid and renal luciferase control plasmid were co-transfected with renal cancer cells with knockdown or overexpression of FOXM1 and ZIC2. 24 hours after transfection, luciferase activity was obtained by using a dual-luciferase reporter assay system (Promega, USA).

### Animal experiments

A subcutaneous xenograft model was established by subcutaneously injecting nude mice with 1 × 10^6^ cells on the left side. The tumor volume was measured with calipers and repeatedly measured every 7 days (length × width^2^)/2. At 28 days following implantation, the mice were euthanized by cervical dislocation. Then, the xenografts were removed, fixed, weighed, photographed, and preserved. For the lung metastasis model, 4×10^6^ tumor cells were injected intravenously. On day 56 after tumor inoculation, mice were sacrificed and lungs were excised, and metastatic nodules were quantified.

### Bioinformatics analysis

All the data were analyzed using the R 3.6.1 software. The batch effect of different data sets was removed by the “SVA” package. Differentially expressed genes (DEGs) analysis was performed by using the “Limma” package. Genes with a False Discovery Rate (FDR) < 0.05 and | log2 (FC) | > 1 were defined as DEGs. Stata15.1 software was used to perform the meta-analysis and complete the forest plot. Gene set enrichment analysis (GSEA 4.2.3) software was used to find the differences between the high- and low-ZIC2 groups in the Hallmark and KEGG pathway (https://www.gsea-msigdb.org/gsea/index.jsp). MethPrime online platform (http://www.urogene.org/methprimer/) was used to design primers for methylation-specific PCR. UCSC Genome Browser (http://genome.ucsc.edu/index.html) was used to visualize H3K4me3 modification, transcription factor binding sites and Chip-seq in the promoter regions. The UCSC Genome Browser includes H3K4me3 modification data for the following cell lines: GM12878, H1-hESC, HSMM, HUVEC, K562, NHEK, and NHLF. AnimalTFDB (http://bioinfo.life.hust.edu.cn/AnimalTFDB/#!/) and GTRD (http://gtrd20-06.biouml.org/) were used to predict transcription factors that might bind to target gene promoters. JASPAR database (https://jaspar.genereg.net/) shows the binding motifs and binding sequences of transcription factors. The cBioPortal for ccRCC genomics was used to explore the genetic variation of ZIC2. The mexpress (https://mexpress.be/) visualized DNA methylation of ZIC2 of TCGA dataset.

### Statistical analysis

The Fisher's exact test or Chi‐squared test was applied for categorical variables, and the Wilcoxon rank-sum test, independent samples t-test or paired samples t-test for continuous variables. Kaplan-Meier curves were plotted, and a log-rank test was used to check the significant difference in overall survival (OS), metastasis-free survival (MFS), and progression-free survival (PFS). The Receiver Operating Characteristic (ROC) analysis was used to examine the sensitivity and specificity of survival prediction. An area under the ROC curve (AUC) served as an indicator of prognostic accuracy. Univariate and multivariate analysis by COX regression show the independent prognostic factors. Pearson correlation test was used to evaluate the correlation between the two groups of data. Two-sided P values less than 0.05 were considered statistically significant for the whole statistical analyses.

## Results

### Oncogenic role and prognosis value of ZIC2 in ccRCC

**Figure 1:**
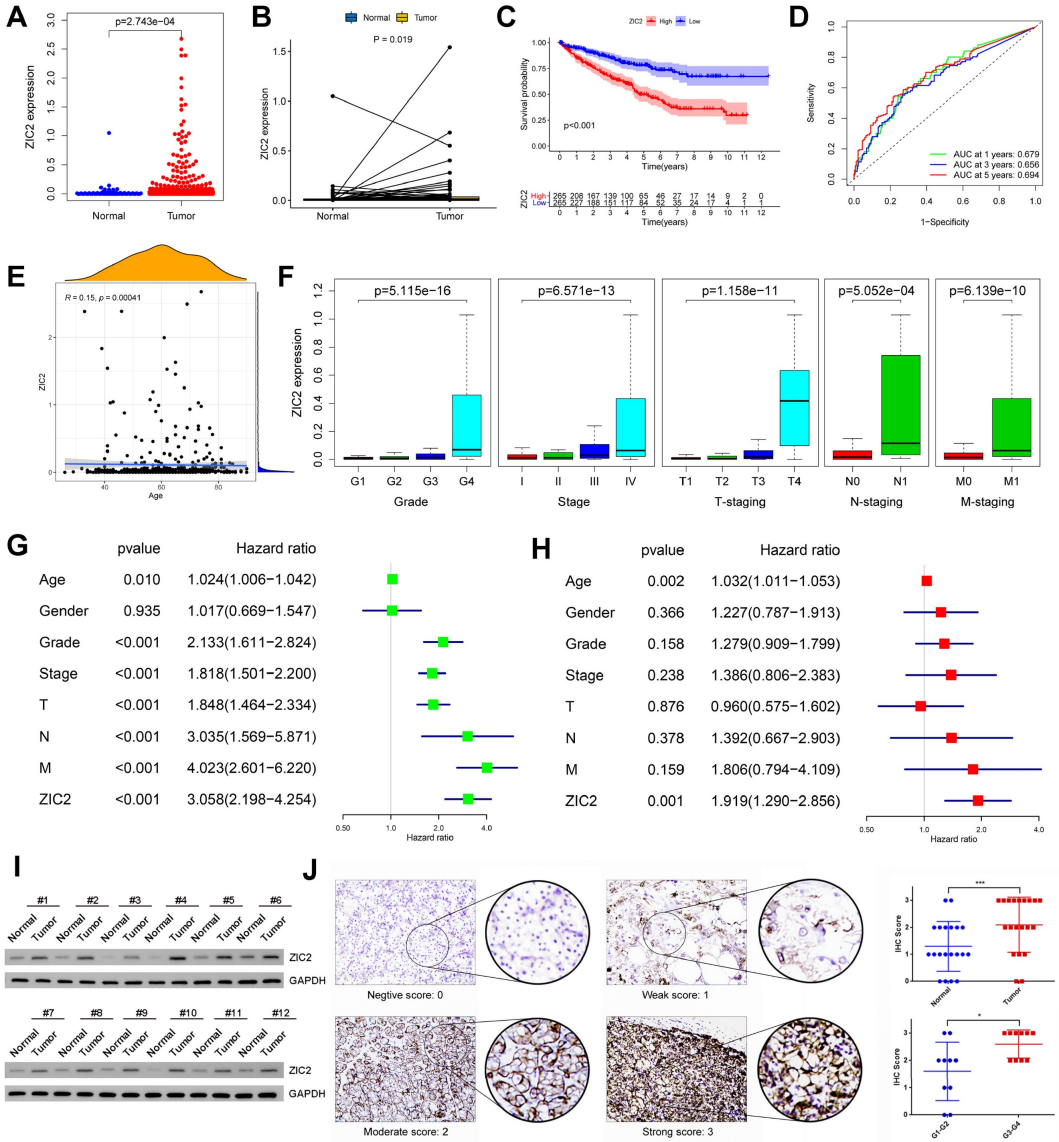
Oncogenic role and prognosis value of ZIC2 upregulation in ccRCC. **(A)** The independent sample t test of ZIC2 in tumor and normal tissues. **(B)** The paired sample t test of ZIC2 in tumor and normal tissues.** (C)** K-M curve analysis of ZIC2 in tumor. **(D)** ROC curve analysis of 1-, 3-, 5-year OS of ZIC2 in tumor. **(E)** Positive correlation between ZIC2 and onset age. **(F)** ZIC2 was significantly correlated with clinicopathological parameters. **(G)** Univariate Cox regression analysis of ZIC2 and clinicopathological parameters. **(H)** Multivariate Cox regression analysis of ZIC2 and clinicopathological parameters. **(I)** Western blot analysis of ZIC2 expression levels in 12 pairs of tumor and corresponding normal tissues. **(J)** Immunohistochemical analysis of ZIC2 expression in 20 pairs of ccRCC tumor tissues and corresponding normal renal tissues.

We analyzed the prognostic role of ZIC2 in ccRCC based on TCGA database. ZIC2 was significantly overexpressed in tumors compared to normal tissues, either based on overall differences or pair-based differences (P < 0.05) (Figure 1A-1B). Taking the median expression value of ZIC2 as the cut-off value, the overall survival performance of patients in the high expression group was better than that in the low expression group (P < 0.05) (Figure 1C). Using the expression of ZIC2 as a predictor, the AUC of 1-, 3-, and 5-year survival was 0.679, 0.656, and 0.694, respectively (Figure 1D). Next, we explored the relationship between the expression of ZIC2 and clinicopathological features and found that the expression of ZIC2 was positively correlated with the age of onset (Figure 1E), and gradually increased with the increase of tumor grade and stage (P < 0.05) (Figure 1F). Finally, univariate and multivariate Cox regression analysis showed that ZIC2 expression was an independent predictor of ccRCC (P < 0.05) (Figure 1G-1H). Concordantly, local samples of ccRCC and adjacent tissues were detected by western blot assay, which also showed up-regulated expression of ZIC2 in ccRCC tissues (Figure 1I). Immunofluorescence also showed ZIC2 expression was indeed higher in the ccRCC tumor tissue than in the adjacent normal tissue. ZIC2 expression was also higher in high-grade tumors (G3-G4) than in low-grade tumors (G1-G2) (Figure 1J).

To avoid the accidental results of a single data set, we collected multiple GEO data sets (GSE781, GSE6344, GSE14762, GSE17895, GSE36895, GSE40435, GSE46699, GSE53000, GSE53757, GSE66272, GSE68417, GSE71963, GSE105261, and GSE126964) to explore the expression differences between tumor and normal tissues. After correcting for multiple sets of data, the pooled meta-analysis forest plot showed that ZIC2 was indeed highly expressed in tumors compared to normal tissues (Figure S1A). Moreover, we also collected complete clinicopathological data sets from these databases and showed that ZIC2 expression increased with tumor stage, Fuhrman grade, pathological grade, and T stage (Figure S1B-S1E). In addition, we downloaded the E-MTAB-1980 dataset with long-term follow-up survival data from the ArrayExpress database. The results showed that regardless of OS or MFS as the clinical observation end point, the prognosis of the ZIC2 high expression group was significantly worse than that of the low expression group (P < 0.05) (Figure S1F-S1G). Similarly, its expression was positively correlated with Fuhrman grading and T staging (Figure S1H).

### ZIC2 promotes proliferation, migration, invasion, epithelial-mesenchymal transition (EMT) and affects the cell cycle of ccRCC cells *in vitro* and *in vivo*

**Figure 2:**
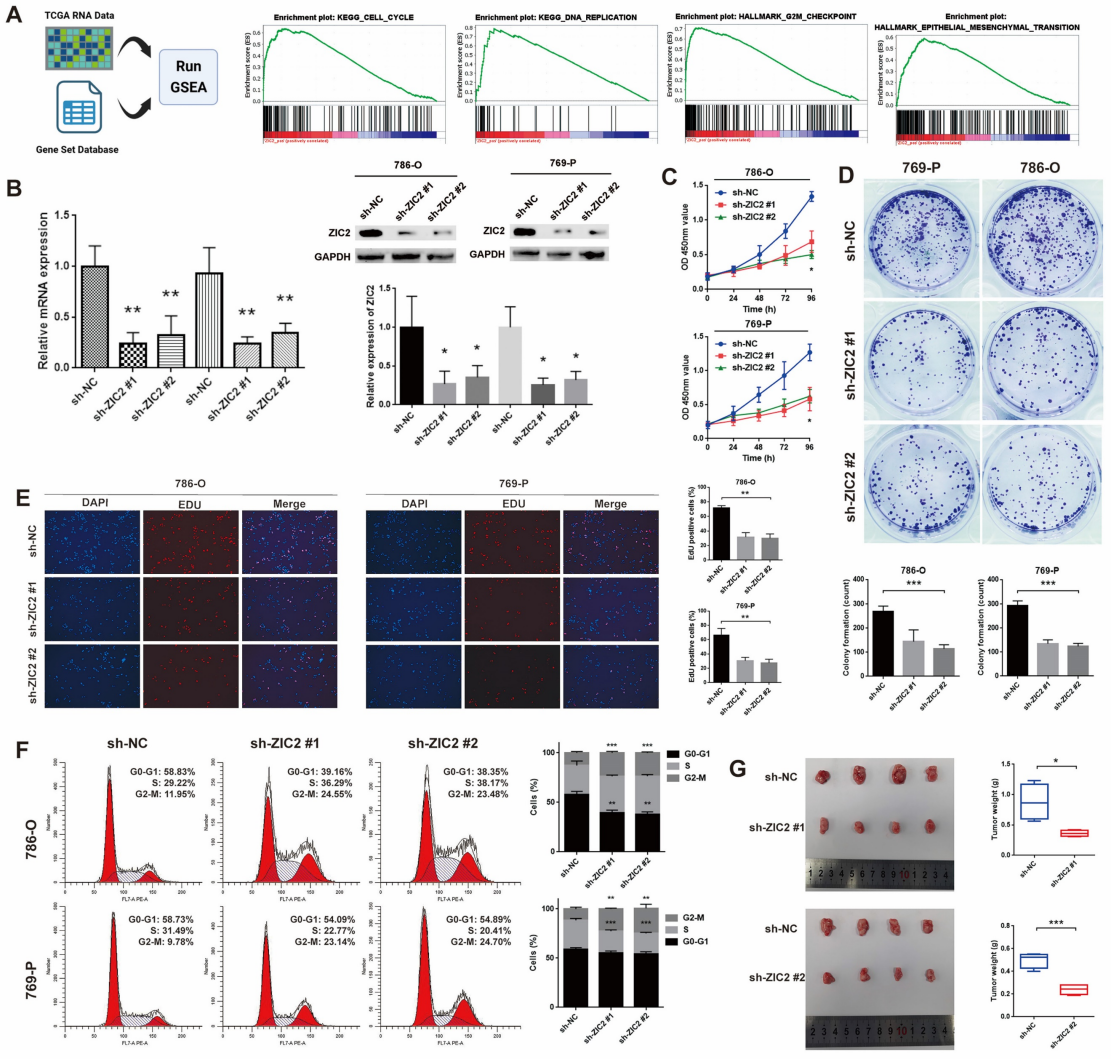
ZIC2 promotes proliferation and affects the cell cycle of ccRCC cells. **(A)** GSEA between the low and high ZIC2 expression groups in the Hallmark and KEGG pathway. **(B)** RT-qPCR and Western blot experiment confirmed the success of ZIC2 knockdown. **(C-E)** When ZIC2 was knocked down, the proliferation and colony forming ability decreased significantly.** (F)** Cell cycle distribution when ZIC2 was knocked down. **(G)** Subcutaneous tumor formation ability was reduced in nude mice when ZIC2 was knocked down.

To assess the biological function of ZIC2 in ccRCC, GSEA was utilized to track down the differences between the low and high ZIC2 expression of TCGA groups in the Hallmark and KEGG pathway. The results showed that the high expression of ZIC2 were significantly enriched in “KEGG CELL CYCLE”, “KEGG DNA REPLICATION”, “HALLMARK G2M CHECKPOINT”, and “HALLMARK EPITHELIAL MESENCHYMAL TRANSITION” (Figure 2A). Therefore, we hypothesized that ZIC2 might affect the proliferation migration, invasion, EMT and affects the cell cycle. To confirm the biological role of ZIC2, we carried out basic experiments. Firstly, we detected the expression level of ZIC2 in different kinds of renal cell carcinoma cell lines. We found that the mRNA and protein expression of ZIC2 in 786-O and 769-P was the highest, and Caki-1 was lowest (Figure S2A). Therefore, we selected these three cell lines for subsequent loss- and gain-of-function experiments. RT-qPCR and Western blot experiment confirmed the success of ZIC2 knockdown and over-expression (Figure 2B and Figure S2B). When ZIC2 was knocked down, the CCK-8 assay, colony formation assay, and EDU assay showed tumor activity, clonogenesis and proliferation were significantly decreased (Figure 2C-2E). Conversely, overexpression of ZIC2 significantly promoted cell growth in Caki-1 cells (Figure S2C-S2E). In addition, compared with the control, ZIC2 knockdown decreased the proportion of G0/G1 phase cells, decreased the proportion of S phase cells, and increased the proportion of G2/M phase cells, indicating that depletion of ZIC2 led to G2/M block (Figure 2F). However, the opposite result was shown after transfection with overexpressed vector (Figure S2F). *In vivo* experiments showed that ZIC2 silencing resulted in tumor growth inhibition (Figure 2G), and overexpression of ZIC2 facilitated tumor growth (Figure S2G).

**Figure 3:**
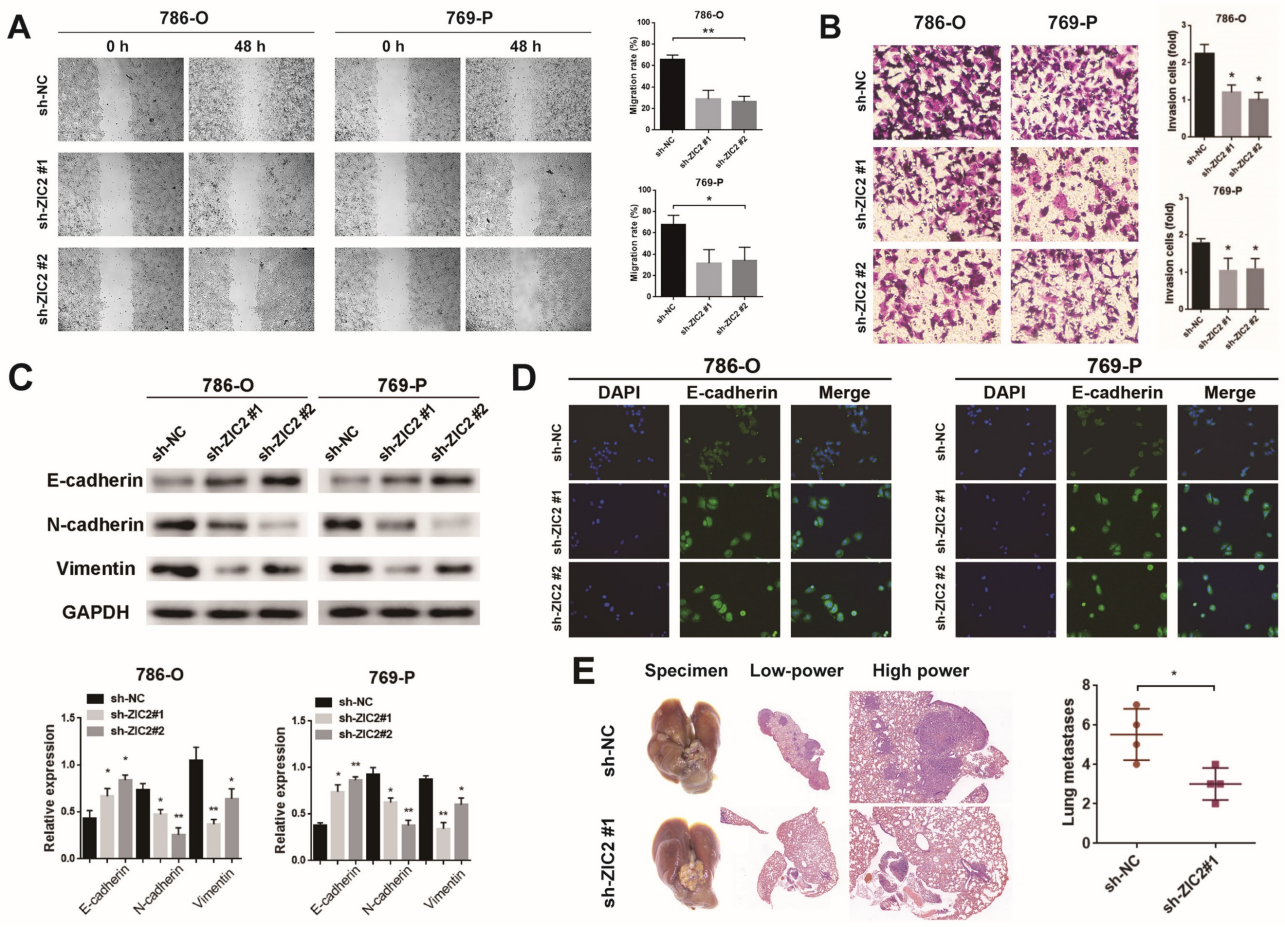
ZIC2 promotes migration, invasion, EMT, and pulmonary metastatic capacity of ccRCC cells. The results of **(A)** wound healing assay, **(B)** invasion chamber assay and **(C)** EMT-specific protein detection after ZIC2 knockdown. **(D)** Cell morphology was determined by immunofluorescence of E-cadherin after ZIC2 knockdown. (E) Knockdown of ZIC2 significantly reduced lung metastasis.

The wound-healing and Transwell assays showed that ZIC2 knockdown significantly decreased cell migrative and invasive abilities in 786-O and 769-P cells and upregulation of ZIC2 enhanced migratory and invasive abilities to Caki-1 cells (Figure 3A-3B and Figure S3A-S3B). Next, we explored whether ZIC2 was associated with EMT of ccRCC cells. Results showed that ZIC2 shRNA decreased the protein levels of N-cadherin and Vimentin expression but increased the E-cadherin expression (Figure 3C). The enhanced ZIC2 expression had an opposite effect on the protein expression of E-cadherin, N-cadherin, and Vimentin (Figure S3C). Immunofluorescence assay of E-cadherin not only reconfirmed that ZIC2 shRNA increased the expression of E-cadherin, but also observed that the cell morphology changed from spindle-like to cobblestone-like (Figure 3D). Overexpression of ZIC2 resulted in the opposite result (Figure S3D). Lung metastasis models were used to investigate the effect of ZIC2 on metastasis. ZIC2 knockdown significantly reduced the number of pulmonary metastasis nodules, while overexpression of ZIC2 promoted the ability of lung metastasis through H&E staining (Figure 3E and Figure S3E). All up, we demonstrated that ZIC2 can promote proliferation, invasion, migration, EMT, and regulate the cell cycle of ccRCC cells.

### Exploration of genetic and epigenetic mechanisms of ZIC2 upregulation

We attempted to explore the molecular mechanism of ZIC2 upregulation. First, the inactivation of the Von Hippel-Lindau (VHL) is a typical hallmark of ccRCC. All TCGA patients were divided into wild type and mutant type according to VHL mutation status (Figure S4A). However, there was no difference in the expression of ZIC2 between the two groups, suggesting that the upregulation of ZIC2 was not related to the VHL mutation (Figure S4B). Next, we explored whether it was related to structural variants, mutations, and copy number variations of ZIC2 gene itself. However, based on five independent data sets, there were only three cases (0.56%) with copy number amplification of ZIC2 in TCGA data set (Figure S4C). The above results indicate that upregulation of ZIC2 is not associated with genetic disorders in ccRCC.

**Figure 4:**
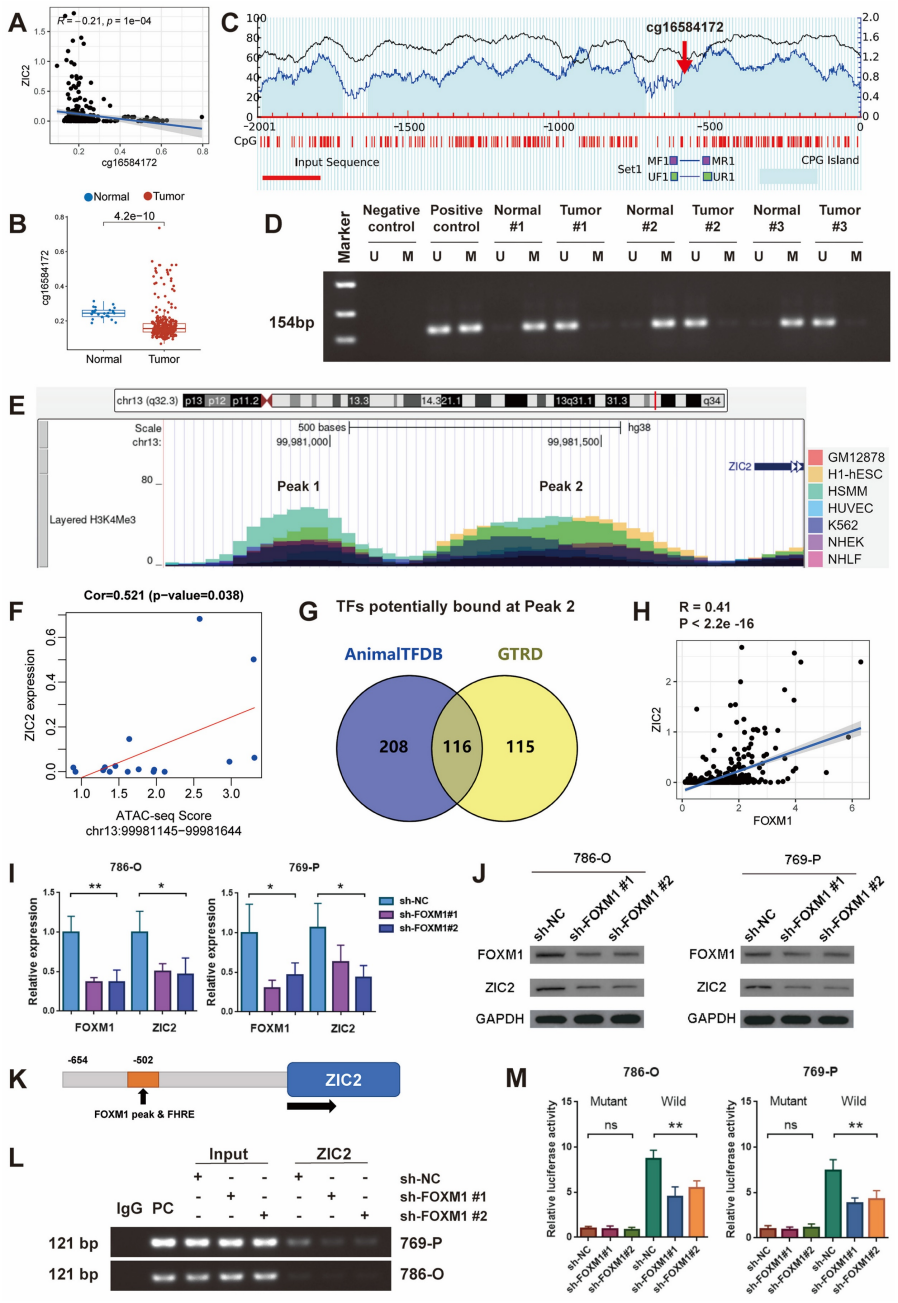
ZIC2 promoter hypomethylation and high H3K4Me3 levels is transcriptically regulated by FOXM1. **(A)** cg16584172 hypomethylation was associated with ZIC2 overexpression. **(B)** Methylation difference of cg16584172 between tumor and normal tissues **(C)** Methylation and non-methylation primers were designed near cg16584172. **(D)** Methylation-specific PCR was used to analyze the difference in methylation levels between the 3 pairs of tumors and the corresponding normal tissues.** (E)** There were two H3K4Me3 enrichment peaks upstream of ZIC2 initiation transcription site.** (F)** There was a significant positive correlation between ATAC-seq score of peak 2 and ZIC2 expression. **(G)** Animal TFDB and GTRD databases predict transcription factors for potential binding to ZIC2 promoters. **(H)** There was a strong correlation between FOXM1 and ZIC2 expression. **(I-J)** Knockdown of FOXM1 significantly inhibited ZIC2 expression at both transcriptional and translational levels. **(K)** The potential binding sites of ZIC2 were identified based on the FOXM1 binding motif. **(L)** Chip-PCR confirmed the binding of FOXM1 to the ZIC2 promoter. **(M)** Luciferase reporter experiments confirmed the ZIC2 binding site sequence by FOXM1.

In addition to genetic variation, gene expression is closely related to epigenetics, such as DNA methylation. We looked at the Methylation profile of ZIC2 genome based on TCGA Infinium Human Methylation 450K data and found that the methylation level of cg16584172 in the promoter region of ZIC2 was significantly negatively correlated with the expression level of ZIC2 gene (Figure S4D and Figure 4A). The methylation level of this site in ccRCC tumor tissues was significantly lower than that in normal tissues (Figure 4B). To test this, we used methylation-specific PCR to detect the methylation of tumor and paracancerous tissues. MethPrimer was used to design methylation-specific and unmethylation-specific primers for the cg16584172 site (Figure 4C). The results showed that ZIC2 was hypomethylated in the tumor tissues and hypermethylated in the adjacent tissues, which confirmed that the high expression of ZIC2 may be related to the hypomethylation of its promoter (Figure 4D).

Histone modification is another type of epigenetic regulation, especially H3K4me3 is often associated with transcriptional activation of nearby genes. We scanned the level of H3K4Me3 in the ZIC2 promoter region through UCSC genome and found that two peaks of H3K4Me3 were prevalent in seven different cell lines: GM12878, H1-hESC, HSMM, HUVEC, K562, NHEK, and NHLF (Peak 1 and Peak 2) (Figure 4E). And then, we employed the Assay for transposase-accessible chromatin using sequencing (ATAC-seq) data of the TCGA dataset further demonstrated that the ATAC-seq peak intensity of peak 2 but not peak 1 was significantly positively correlated with ZIC2 expression (Figure 4F and Figure S4E).

### ZIC2 is positively transcriptionally regulated by the transcription factor FOXM1

Regardless of the presence of DNA hypomethylation, high H3K4Me3 and chromatin opening in the promoter region, all contribute to the binding of transcription factors to initiate transcription. Therefore, we searched Animal TFDB and GTRD databases for transcription factors that could potentially bind to the ZIC2 promoter region and found 116 candidate transcription factors (Figure 4G). Among them, we found the strongest positive correlation between FOXM1 and ZIC2 expression (Figure S5A and Figure 4H). It is worth mentioning that FOXM1 is significantly higher expressed in tumors than in normal tissues (Figure S5B), and the expression increases with the increase of tumor stage (Figure S5C). High expression of FOXM1 was associated with worse OS and progression-free survival (Figure S5D-S5E). We then knocked down FOXM1 expression with shRNA and found that ZIC2 expression was down-regulated simultaneously at both the transcriptional and protein levels (Figure 4I-4J). Conversely, overexpression of FOXM1 was accompanied by overexpression of ZIC2 (Figure S5F-S5G). We searched the Cistrome Data Browser and found that chip-seq targeting FOXM1 did find an enrichment peak in the promoter region of ZIC2 (Figure S5H). We employed JASPAR to identify a potential binding motif for FOXM1 located 502 bp upstream of the transcription start site of ZIC2 (Figure 4K). Based on this binding site, PCR primers were designed, and Chip-PCR showed that FOXM1 indeed directly bound to promoter DNA of ZIC2 (Figure 4L). And the PCR products increased and decreased simultaneously with the up-regulation and down-regulation of FOXM1 (Figure 4L and Figure S5I). The promoter region containing this motif and the luciferase gene were used to construct a luciferase reporter system (Figure S5J). The results showed that knockdown of FOXM1 significantly reduced the fluorescence intensity of wild type but had no effect on mutant type, which showed lower fluorescence intensity (Figure 4M). In contrast, overexpression of FOXM1 significantly induced the wild-type reporter luciferase activity but not the mutant reporter luciferase activity (Figure S5K). The above results confirmed that FOXM1 was able to directly bind to the promoter region of ZIC2 to promote ZIC2 expression.

### ZIC2 up-regulates UBE2C expression and activates AKT/mTOR signaling pathway

**Figure 5:**
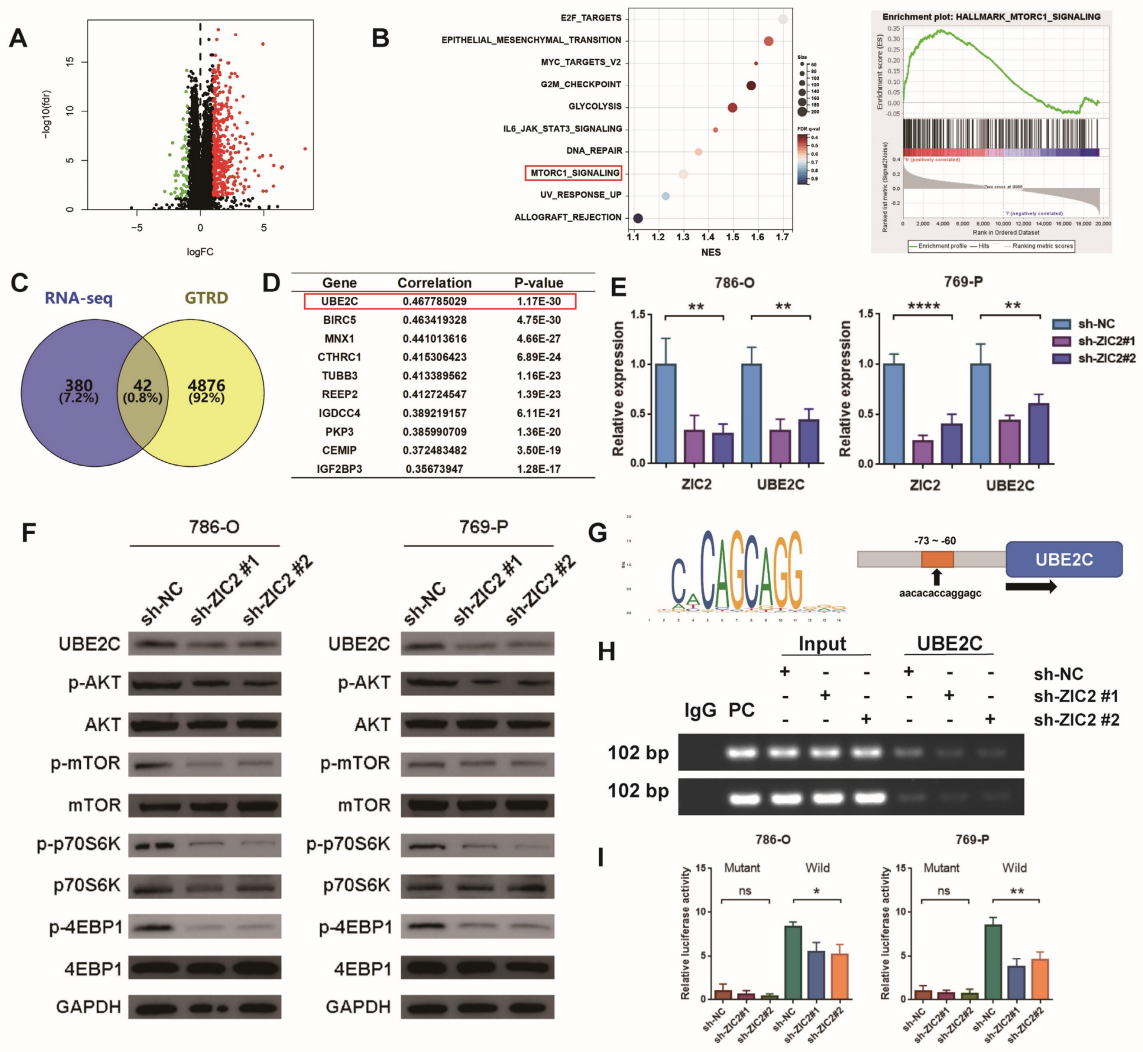
ZIC2 up-regulates UBE2C expression and activates AKT/mTOR signaling pathway. **(A)** Differentially expressed between high- and low-expression ZIC2 in TCGA dataset. **(B)** GSEA analysis for signaling pathways enriched in highly expressing ZIC2. **(C)** The intersection of the high ZIC2 expression -induced gene and the potential target gene of ZIC2.** (D)** List of genes most associated with ZIC2 expression. **(E)** Knockdown of ZIC2 significantly inhibited UBE2C expression. **(F)** ZIC2 knockdown significantly inhibited UBE2C protein expression and AKT/mTOR signaling pathway activation. **(G)** Transcriptional binding motifs of ZIC2 and potential binding sites to UBE2C. **(H)** Chip-PCR confirmed the binding of ZIC2 to the UBE2C promoter. **(I)** Luciferase reporter experiments confirmed the UBE2C binding site sequence by ZIC2.

We tried to explore the downstream regulatory mechanism of ZIC2 promoting the malignant phenotype of ccRCC. First, we compared the transcriptome sequencing of high-expression ZIC2 and low-expression ZIC2 in TCGA dataset and found that 422 genes were up-regulated and 51 genes were down-regulated (Figure 5A). GSEA analysis showed that the high-expression ZIC2 were significantly enriched in the “MTORC1_signaling” pathways compared with the low-expression ZIC2 (Figure 5B). Western blot assays confirmed that ZIC2 knockdown significantly reduced the phosphorylation of AKT and mTOR, as well as the phosphorylation of downstream effector factors (p70S6K and 4EBP1) (Figure 5F), whereas ZIC2 overexpression showed the opposite results (Figure S6F). As to how ZIC2 activates the AKT/mTOR signaling pathway, we considered that ZIC2 itself acts as a transcription factor that may transactivate some key genes. So, the genes up-regulated by ZIC2 overexpression were collected and intersected with the target genes predicted by GTRD database, which suggested 42 potential candidates (Figure 5C). Among them, UBE2C showed the strongest co-expression with ZIC2, which aroused our great interest (Figure 5D). Data from other datasets (GSE23629, GSE73731, and GSE150404) similarly showed a strong correlation between UBE2C and ZIC2 expression (Figure S6A). Previous studies have reported that UBE2C can promote tumor proliferation by activating AKT/mTOR signaling pathway 12. It is worth mentioning that UBE2C is also highly expressed in tumors (Figure S6B), high expression of UBE2C is also associated with worse OS (Figure S6C), and higher pathological stage is associated with higher UBE2C expression (Figure S6D). After ZIC2 knockdown or overexpression, UBE2C showed simultaneous down-regulation or up-regulation at both mRNA and protein levels (Figure 5E-5F and Figure S6F-S6G). The above results all highly suspect that ZIC2 is a transcription factor targeting UBE2C. To prove this, we found that Chip-seq targeting ZIC2 had significant enrichment peaks in the promoter region of UBE2C (Figure S6E), and JASPAR database also predicted the potential binding sites between ZIC2 and the promoter region of UBE2C. The binding motifs of ZIC2 target the binding site of UBE2C about 73~60bp upstream of the UBE2C initiation transcription site (Figure 5G). Based on this, we designed PCR primers for Chip-PCR experiment. It was indeed found that the target product was amplified by immunoprecipitation of ZIC2 antibody, and the amplified DNA fragment product decreased or increased simultaneously with ZIC2 knockdown or overexpression (Figure 5H and Figure S6H). In order to prove more accurately, we carried out luciferase reporting experiments by mutating binding sites (Figure S6I) and found that the luciferase activity of the mutant type was significantly reduced compared with that of the wild type. In the wild-type luciferase reporting system, luciferase activity was simultaneously inhibited or promoted with the intervention of ZIC2 expression (Figure 5I and Figure S6J). The above results together prove that ZIC2, as a transcription activator, regulates UBE2C expression positively.

### UBE2C is required for ZIC2-induced AKT/mTOR signaling activation and ccRCC malignant phenotype

**Figure 6:**
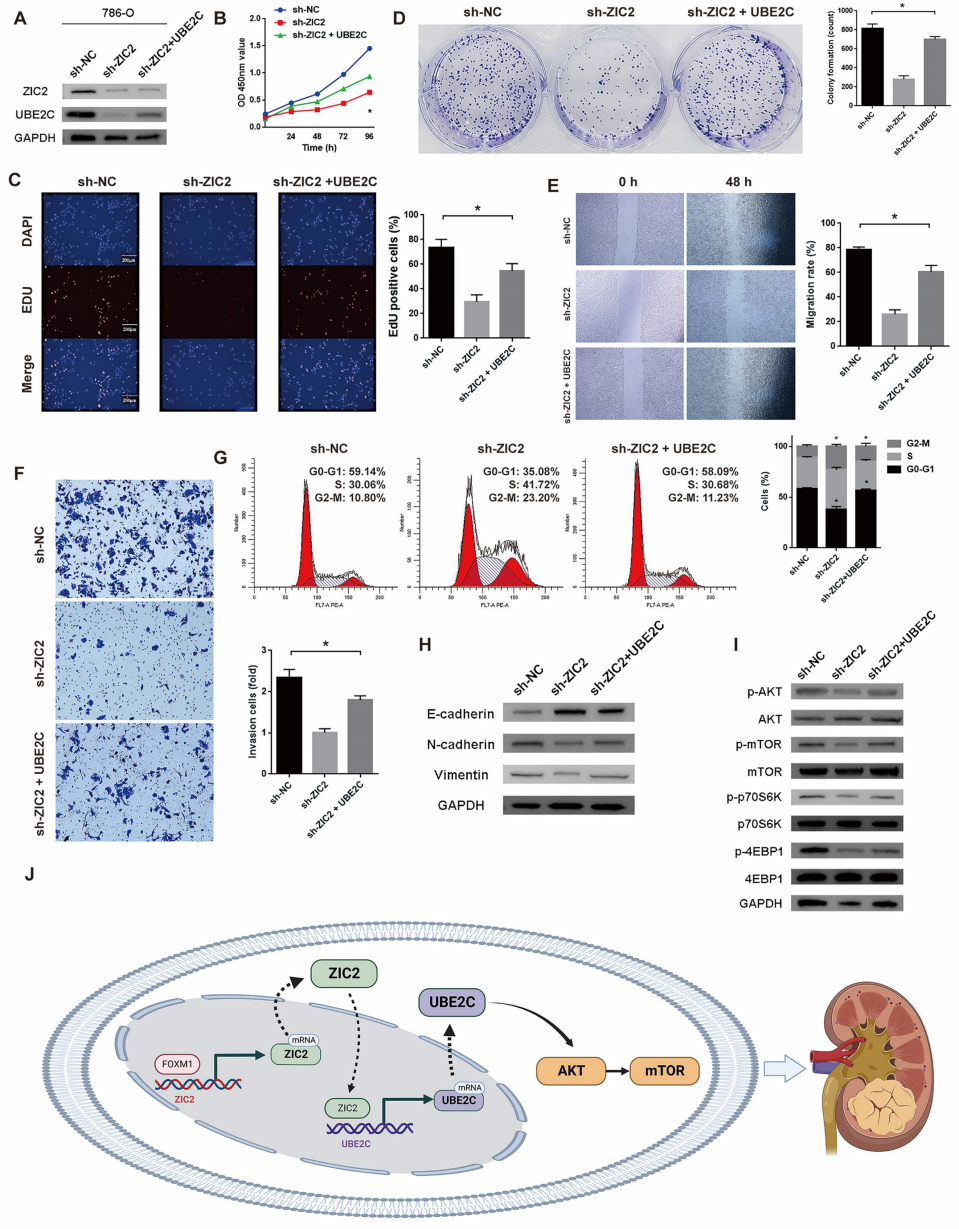
UBE2C is required for ZIC2-induced AKT/mTOR signaling activation and ccRCC malignant phenotype. **(A)** UBE2C was overexpressed on the basis of ZIC2 knockdown. Overexpression of UBE2C partially rescued the decreased **(B-C)** proliferation ability, **(D)** colony formation ability, **(E)** migration, **(F)** invasion and **(H)** EMT ability caused by ZIC2 knockdown, and also partially rescued **(G)** the G2/M cell cycle arrest and **(I)** AKT/mTOR signaling pathway inhibition.** (J)** Schematic of the mechanism of this study.

Above, we only proved that ZIC2 can regulate the expression of UBE2C, but it is unclear whether ZIC2 activates AKT/mTOR signaling pathway and promotes tumorigenesis through the participation of UBE2C. Therefore, we overexpressed UBE2C in the ZIC2 knockdown 786-O cell line (Figure 6A). The following series of phenotypic experiments showed that overexpression of UBE2C significantly rescued the decrease of tumor activity, cloning formation, proliferation, invasion, and migration caused by ZIC2 knockdown (Figure 6B-6F). Meanwhile, overexpression of UBE2C significantly rescued the cell cycle G2/M arrest induced by ZIC2 knockdown (Figure 6G). ZIC2 knockdown induced EMT was also rescued by UBE2C overexpression (Figure 6H). Moreover, western blot analysis showed that UBE2C overexpression significantly reversed the inhibition of AKT/mTOR signaling pathway caused by ZIC2 knockdown (Figure 6I). The above results revealed that UBE2C is required for ZIC2-induced AKT/mTOR signaling activation and ccRCC malignant phenotype. Finally, the overall mechanism of this study is shown in Figure 6J, which demonstrates that FOXM1-ZIC2-UBE2C-AKT/mTOR signaling promotes the malignant phenotype of ccRCC.

## Discussion

In this study, we systematically analyzed the ZIC2 gene in ccRCC and found that the expression of ZIC2 in ccRCC was higher. High expression of ZIC2 predicted poor prognosis. *In vivo* and *in vitro* experiments confirmed that ZIC2 regulated cell cycle and promoted cell proliferation, invasion, and metastasis of renal cancer cells. Its upstream is transcriptionally regulated by FOXM1, and its downstream regulates UBE2C through positive transcription, thereby activating AKT/mTOR signaling pathway. This implies that ZIC2 may be an oncogenic regulator and has the potential to be a new target for the treatment of ccRCC.

The ZIC family, which consists of five members including ZIC2, are vertebrate homologues of the Drosophila odd-paired gene and encode zinc finger transcription factor, which are known to be involved in a variety of tumor diseases, including bladder cancer 13, melanoma 14, hepatocellular carcinoma 15, epithelial ovarian tumor 16, malignant pleural mesothelioma 17, liposarcomas 18 and so on. As the representative transcription factor of ZIC family, the significant effect of ZIC2 in Pan cancer has been widely studied, which is a focal point having been paid close attention to by our study group 9.

Regarding the regulatory mechanism of ZIC2, relevant studies have been explored. LINC00200 binds to the RNA-binding protein IGF2BP3 to enhance ZIC2 mRNA stability in neuroblastoma 19. circDPP4 acted as a miR-564 sponge and indirectly regulated the expression of ZIC2 in prostate cancer 20. LINC00665 also served as a sponge for miR-181c-5p and then regulated ZIC2 expression in lung adenocarcinoma 21. miR-873 and miR-129-5p down-regulate ZIC2, and HOXA10 can directly bind to the promoter of ZIC2 and up-regulate ZIC2 transcription in nasopharyngeal carcinoma 22-24. miR-1284, miR-129-5p and miR-1271‑5p can also downregulate ZIC2 expression in breast cancer, prostate cancer, cervical cancer, acute myeloid leukemia and liver cancer, respectively 25-29. Most of the current research focuses on the regulatory mechanisms of non-coding RNAs. In this study, in terms of DNA methylation, histone modification and transcription factor regulation, we found that the ZIC2 promoter region is highly open and subject to the positive transcriptional regulation of FOXM1. The overexpression of FOXM1 has been found to be associated with tumor progression in ccRCC patients 30, 31. FOXM1 promotes ccRCC progression by being phosphorylated, deubiquitinated, or directly transcribed to activate target genes 32-34. This study is the first to show that FOXM1 can directly target the ZIC2 promoter region to turn on positive transcription.

The downstream regulation mechanism of ZIC2 has also been reported. ZIC2 can activate TGF-β, Src/FAK, Notch, Raf/MEK/ERK, and Wnt/β-catenin signaling pathway in colorectal cancer, non-small cell lung cancer, endometrial cancer, liver cancer, and ccRCC cells 28, 35-39. ZIC2 can transcriptional regulates Src, lncRNA SNHG12, Axin2, Runx2, OCT4, STAT3, PAK4 expression in non-small cell lung cancer, endometrial cancer, colon cancer, ccRCC, lung adenocarcinoma, liver cancer, and breast cancer 15, 28, 36-41. In this study, we found that UBE2C is a new downstream target gene of ZIC2. As for UBE2C, studies have confirmed the overexpression of UBE2C in ccRCC tissues by RT-qPCR and IHC analysis. In addition, UBE2C was associated with clinical factors such as TNM stage, gender, and pathological stage 42. Basic experiments showed that it affected the proliferation rate, cell vitality, cell cycle progression, migration rate and invasion rate of ccRCC cells, and was regulated by lncRNA FTX/miR 4429 43.

This study finally found that mTOR signaling is the terminal signaling pathway mechanism that affects the progression of ccRCC. As a classical signaling pathway mechanism for the occurrence and development of renal cell carcinoma, mTOR signaling has been widely studied from biological role to clinical practice 44. Everolimus, an mTOR inhibitor, has antitumor and antiangiogenic activity and has been shown to improve progression-free survival in patients with vascular endothelial growth factor receptor-tyrosine kinase inhibitors 45. This study expands the upstream regulatory mechanism of mTOR signaling, which is regulated by FOXM1-ZIC2-UBE2C axis.

## Conclusions

In conclusion, this study demonstrated that ZIC2 may act as an oncogenic regulator in ccRCC, which is transcriptase-regulated by FOXM1 and directly regulates UBE2C expression to activate mTOR signaling pathway. Our study elucidated the FOXM1/ZIC2/UBE2C/mTOR signaling axis, providing a new molecular mechanism and therapeutic target for RCC development.

## Supplementary Material

Supplementary figures and tables.

## Abbreviations

ZIC2: Zic family member 2
ccRCC: clear cell renal cell carcinoma
MS-PCR: methylation-specific PCR
ChIP: Chromatin immunoprecipitation
OS: overall survival
MFS: metastasis-free survival
PFS: progression-free survival
ROC: Receiver Operating Characteristic
AUC: Area under the curve
EMT: epithelial-mesenchymal transition
GSEA: Gene set enrichment analysis
VHL: Von Hippel-Lindau
ATAC-seq: Assay for transposase-accessible chromatin using sequencing
