# Supplementary material for: FOXM1-regulated ZIC2 promotes the malignant phenotype of renal clear cell carcinoma by activating UBE2C/mTOR signaling pathway
Source: Int J Biol Sci. 2023 Jun 26;19(11):3293–306. doi: 10.7150/ijbs.84067 (PMC10367559; doi:10.7150/ijbs.84067)
Supplement: Supplementary file 1 — Supplementary figures and tables. [file ijbsv19p3293s1.pdf]

FOXM1-regulated ZIC2 promotes the malignant phenotype of renal clear cell carcinoma by activating UBE2C/mTOR signaling pathway

Zhengtong Lv [1, 2], Miao Wang [1], Huimin Hou [1], Guyu Tang [3], Haozhe Xu [4], Xuan Wang [1, 2, *], Yuan Li [4, *], Jianye Wang [1,2, *], Ming Liu [1,2, *]

1. Department of Urology, Beijing Hospital, National Center of Gerontology; Institute of Geriatric Medicine, Chinese Academy of Medical Sciences, P.R. China.
2. Graduate School of Peking Union Medical College and Chinese Academy of Medical Sciences, P.R. China.
3. Department of Urology, Xiangya Hospital, Central South University, Changsha, Hunan, P.R. China.
4. Department of Urology, Second Xiangya Hospital, Central South University, Changsha, Hunan, P.R. China.

* These authors contributed equally.

Corresponding authors: Xuan Wang (wangxuan4443@bjhmoh.cn), Yuan Li (yuanlixy@csu.edu.cn), Jianye Wang (wangjybjh@126.com), and Ming Liu (liumingbjh@126.com).

Table S1 Information for datasets selected in this study.

Accession Number	Platform	Number of Patients	Male (%)	Age (mean ±sd)	Fuhrman (1/2/3/4)	Grade (G1/G2/G3/G4)	Stage (I/II/III/IV)	Survival Data
TCGA-KIRC	Illumina HiSeq 2000 RNA Sequencing V2	530	64.91	60.56± 12.14	NA	14/230/207/78	269/57/125/83	OS
E-MTAB-1980	Agilent Human Gene Expression 4x44K v2 Microarray 026652 G4845A	101	76.24	63.48± 11.50	13/59/22/5	NA	NA	OS/MFS
GSE781	Affymetrix Human Genome U133B Array	12	75	62.33± 7.36	4/2/3/0	NA	NA	NA
GSE6344	Affymetrix Human Genome U133B Array	10	NA	NA	NA	NA	NA	NA
GSE14762	Affymetrix GeneChip Human Genome U133 Plus 2.0 Array [MBNI v6 Entrez Gene ID CDF]	10	NA	NA	NA	NA	NA	NA
GSE17895	Affymetrix GeneChip Human Genome U133 Plus 2.0 Array (MBNI v11 Entrez Gene ID CDF)	138	NA	NA	NA	NA	NA	NA
GSE36895	Affymetrix Human Genome U133 Plus 2.0 Array	29	58.62	63.25± 14.19	2/13/6/8	NA	5/2/3/6	NA
GSE40435	Illumina HumanHT-12 V4.0 expression beadchip	101	58.42	64.11± 9.19	NA	NA	14/27/13/5	NA
GSE46699	Affymetrix Human Genome U133 Plus 2.0 Array	67	NA	NA	NA	NA	NA	NA
GSE53000	Affymetrix Human Gene 1.0 ST Array [transcript (gene) version]	53	64.15	61.66± 9.02	NA	NA	NA	NA
GSE53757	Affymetrix Human Genome U133 Plus 2.0 Array	72	NA	NA	NA	NA	24/19/14/15	NA
GSE66272	Affymetrix Human Genome U133 Plus 2.0 Array	27	70.37	64.22±	NA	1/16/8/2	NA	NA

				9.19				
GSE68417	Affymetrix Human Gene 1.0 ST Array [transcript (gene) version]	29	NA	NA	2/11/1 4/2	NA	NA	NA
GSE71963	Agilent-014850 Whole Human Genome Microarray 4x44K G4112F (Probe Name version)	32	NA	NA	NA	NA	NA	NA
GSE73731	Affymetrix Human Genome U133 Plus 2.0 Array	265	61.07	NA	NA	22/90/95/ 49	41/12/28 /44	NA
GSE105261	Illumina HumanHT-12 V4.0 expression beadchip	9	NA	NA	NA	NA	NA	NA
GSE126964	HiSeq X Ten (Homo sapiens)	55	74.55	58.36± 10.15	NA	4/21/12/3	NA	NA
GSE150404	Affymetrix Human Gene 2.1 ST Array [transcript (gene) version]	60	48.28	62.08± 7.43	NA	NA	15/15/15 /15	NA

Table S2 The target sequence of sh-ZIC2 and sh-FOXM1.

Gene	Target sequence
sh-ZIC2 #1	5’-CCGGCCGGAGTCTTTGAAGCTGAAACTCGAGTTTCAGCTTCAAAGACTCCGGTTTTTG-3’
sh-ZIC2 #2	5’-CCGGGCAACTGAGCAATCCCAAGAACTCGAGTTCTTGGGATTGCTCAGTTGCTTTTTG-3’
sh-FOXM1#1	5′-CCGGGCCCAACAGGAGTCTAATCAACTCGAGTTGATTAGACTCCTGTTGGGCTTTTTG-3’
sh-FOXM1#2	5′-CCGGGCCAATCGTTCTCTGACAGAACTCGAGTTCTGTCAGAGAACGATTGGCTTTTTG-3’

Table S3 PCR primers used in this study.

Target	FW/RV	Sequences
GAPDH	FW	CAGCCGAGCCACATCG
	RV	TGAGGCTGTTGTCATACTTCTC
FOXM1	FW	TGCAGCTAGGGATGTGAATCTTC
	RV	GGAGCCCAGTCCATCAGAACT
ZIC2	FW	CACCTCCGATAAGCCCTATCT
	RV	GGCGTGGACGACTCATAGC
UBE2C	FW	CTGCCTTCCCTGAATCAGACAACC
	RV	TCGGCAGCATGTGTGTTCAAGG
methylation-specific ZIC2	FW	GTAGTTAATGTTAAGCGTCGAGGTC
	RV	ACAAACGAAATCCAAACGTC
unmethylation-specific ZIC2	FW	AGTTAATGTTAAGTGTTGAGGTTGA
	RV	ACAAACAAAATCCAAACATC
Chip-ZIC2 promoter	FW	TTAAAGAAAGGGGGAGCGGC
	RV	CAGTGTTTAGCCCTCCTCGG
Chip-UBE2C promoter	FW	CCCAAGCGAGCCATTGATTG
	RV	GCAGAGAGACAGGAACTCGG

Table S4 Product information of primary antibodies.

**Protein**	**Product codegong**	**Brand**
ZIC2	ab150404	Abcam
FOXM1	13147-1-AP	Proteintech
UBE2C	ab252940	Abcam
GAPDH	60004-1-Ig	Proteintech
E-cadherin	3195	Cell Signaling Technology
N- cadherin	13116	Cell Signaling Technology
Vimentin	5741	Cell Signaling Technology
p-AKT	4060	Cell Signaling Technology
AKT	9272	Cell Signaling Technology
p-mTOR	2971	Cell Signaling Technology
mTOR	2983	Cell Signaling Technology
p-p70S6K	9205	Cell Signaling Technology
p70S6K	9202	Cell Signaling Technology
p-4EBP1	2855	Cell Signaling Technology
4EBP1	9644	Cell Signaling Technology

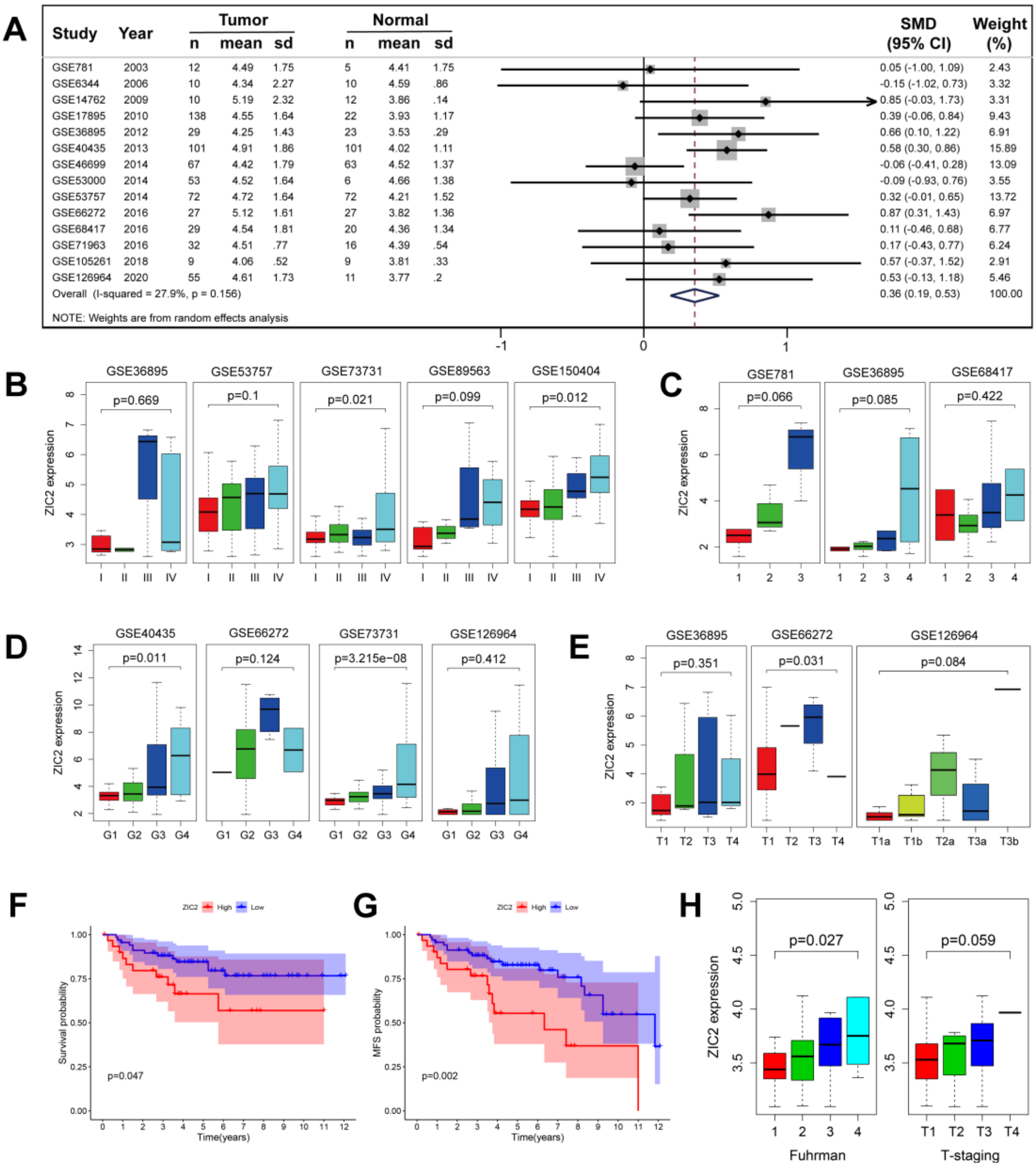

Figure S1: Verification of high expression of ZIC2 and its role in prognosis of ccRCC. **(A)** Meta-analysis of ZIC2 in multiple GEO datasets showed that ZIC2 was highly expressed in tumors. **(B)** The expression of ZIC2 increased with tumor stage. **(C)** The expression of ZIC2 increased with the increase of Fuhrman grade. **(D)** The expression of ZIC2 increased with the pathological grade of tumor. **(E)** The expression of ZIC2 increased with the increase of pathological T stage. **(F-G)** The OS and MFS of high ZIC2 expression group were worse than that of low ZIC2 expression group in E-MTAB-1980 dataset. **(H)** The expression of ZIC2 increased with the increase of Fuhrman grade and T stage.

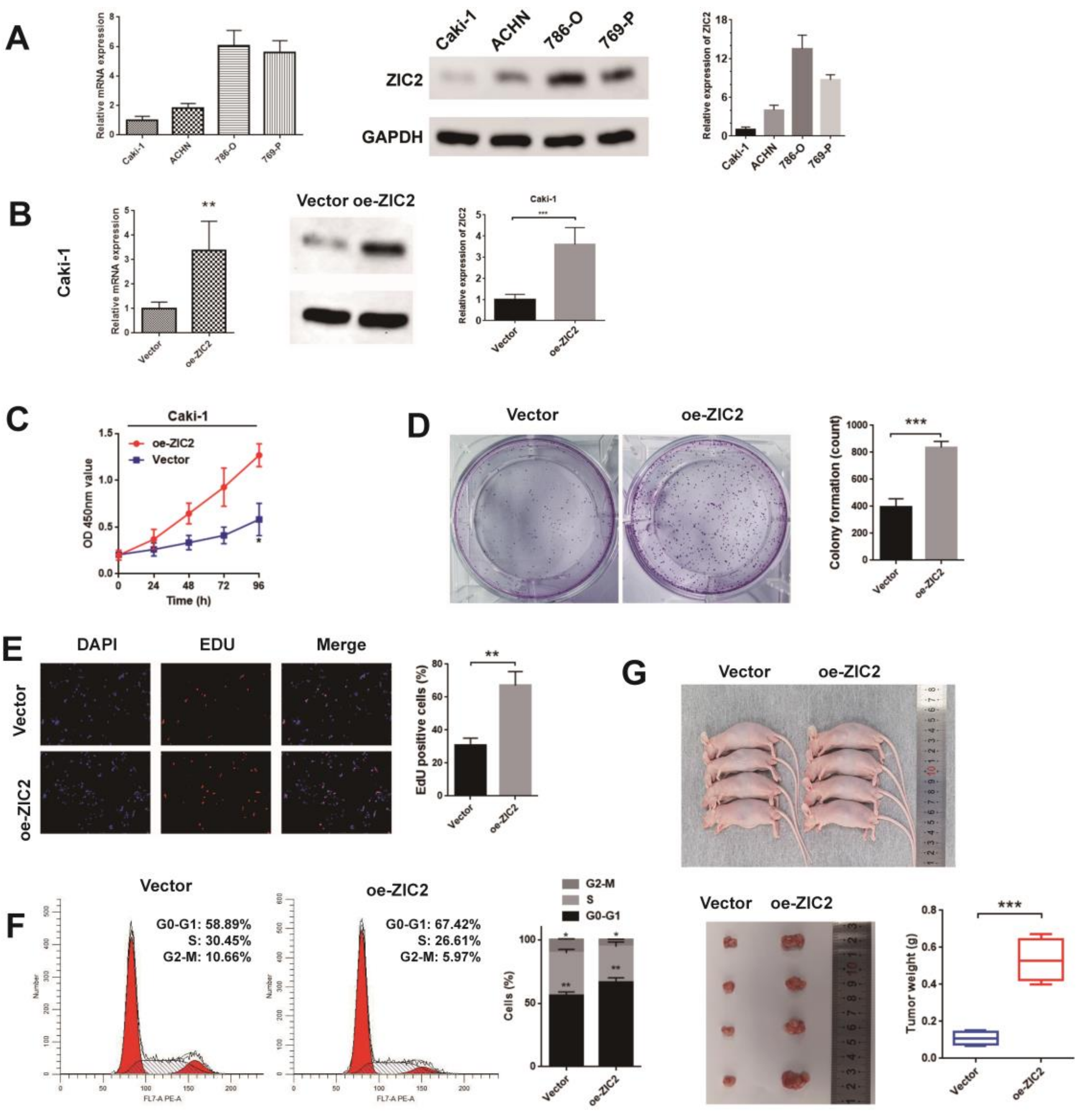

Figure S2: ZIC2 promotes proliferation and affects the cell cycle of ccRCC cells. **(A)** the mRNA and protein expression level of ZIC2 in different kinds of renal cell carcinoma cell lines. **(B)** RT-qPCR and Western blot experiment confirmed the success of ZIC2 overexpression. **(C-E)** When ZIC2 was over-expressed, the proliferation and colony forming ability increased significantly. **(F)** Cell cycle distribution when ZIC2 was over-expressed. **(G)** Subcutaneous tumor formation ability was enhanced in nude mice when ZIC2 was over-expressed.

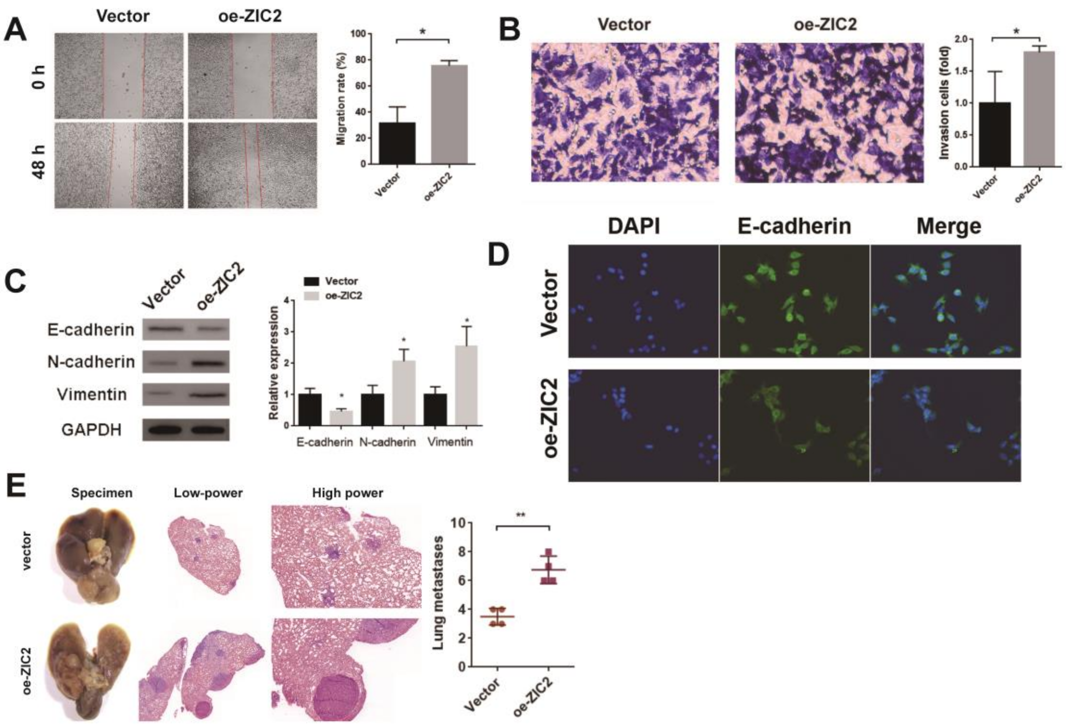

Figure S3: ZIC2 promotes migration, invasion, EMT, and pulmonary metastatic capacity of ccRCC cells. The results of wound healing assay **(A)**, invasion chamber assay **(B)** and EMT-specific protein detection **(C)** after ZIC2 overexpression. **(D)** Cell morphology was determined by immunofluorescence of E-cadherin after ZIC2 overexpression. **(E)** overexpression of ZIC2 significantly promoted lung metastasis.

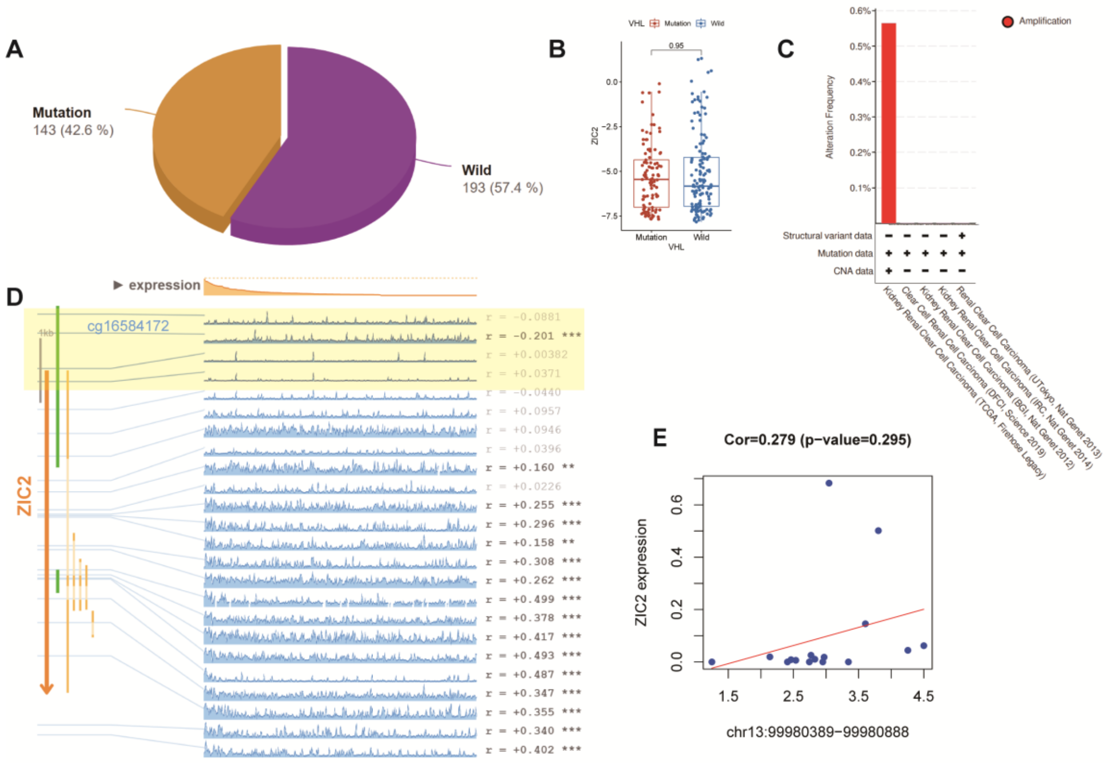

Figure S4: Exploration of genetic and epigenetic mechanisms of ZIC2 upregulation. **(A)** VHL mutation status in the TCGA cohort. **(B)** ZIC2 expression between VHL mutant and wild type. **(C)** Structural variants, mutations, and copy number variations of ZIC2 gene in five independent data sets. **(D)** Methylation status of the ZIC2 gene (methylation sites in the promoter region are shown in yellow background). **(E)** There was no statistical correlation between ATAC-seq score of peak 1 and ZIC2 expression.

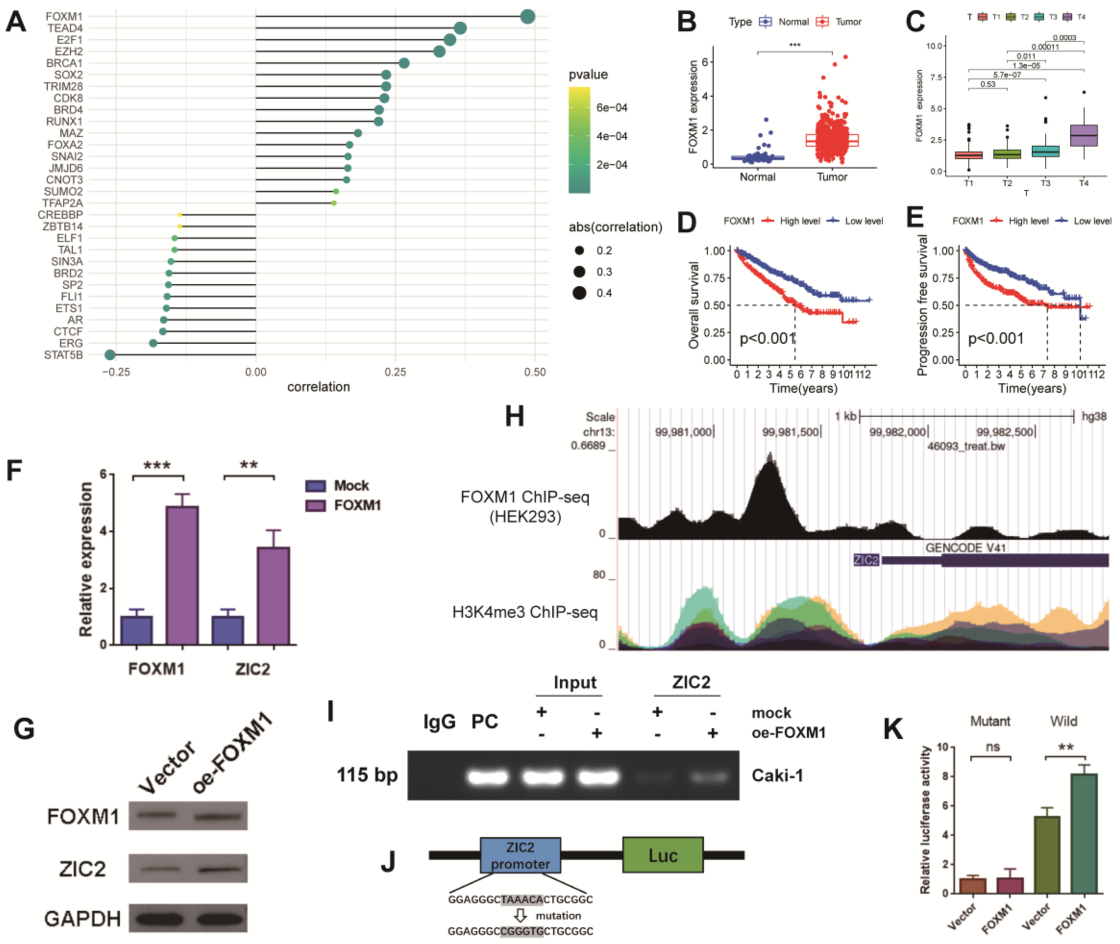

Figure S5: ZIC2 is transcriptically regulated by FOXM1. **(A)** The potential binding transcription factors were further screened by correlation analysis with ZIC2 expression. **(B)** FOXM1 was highly expressed in ccRCC tissues compared with normal tissues. **(C)** The expression of FOXM1 increased with the increase of ccRCC stage. **(D-E)** High expression of FOXM1 was associated with worse OS and PFS. **(F-G)** High expression of FOXM1 was associated with worse OS and PFS. **(H)** Chip-seq targeting FOXM1 revealed a significant sequencing peak upstream of the ZIC2 transcription start site. **(I)** Chip-PCR showed that overexpression of FOXM1 caused an increase in the amplification products to ZIC2 promoter DNA sequence. **(J)** Construction of luciferase reporter system (wild type and mutant type). **(K)** Overexpression of FOXM1 significantly increased the luciferase activity of the wild-type luciferase reporter.

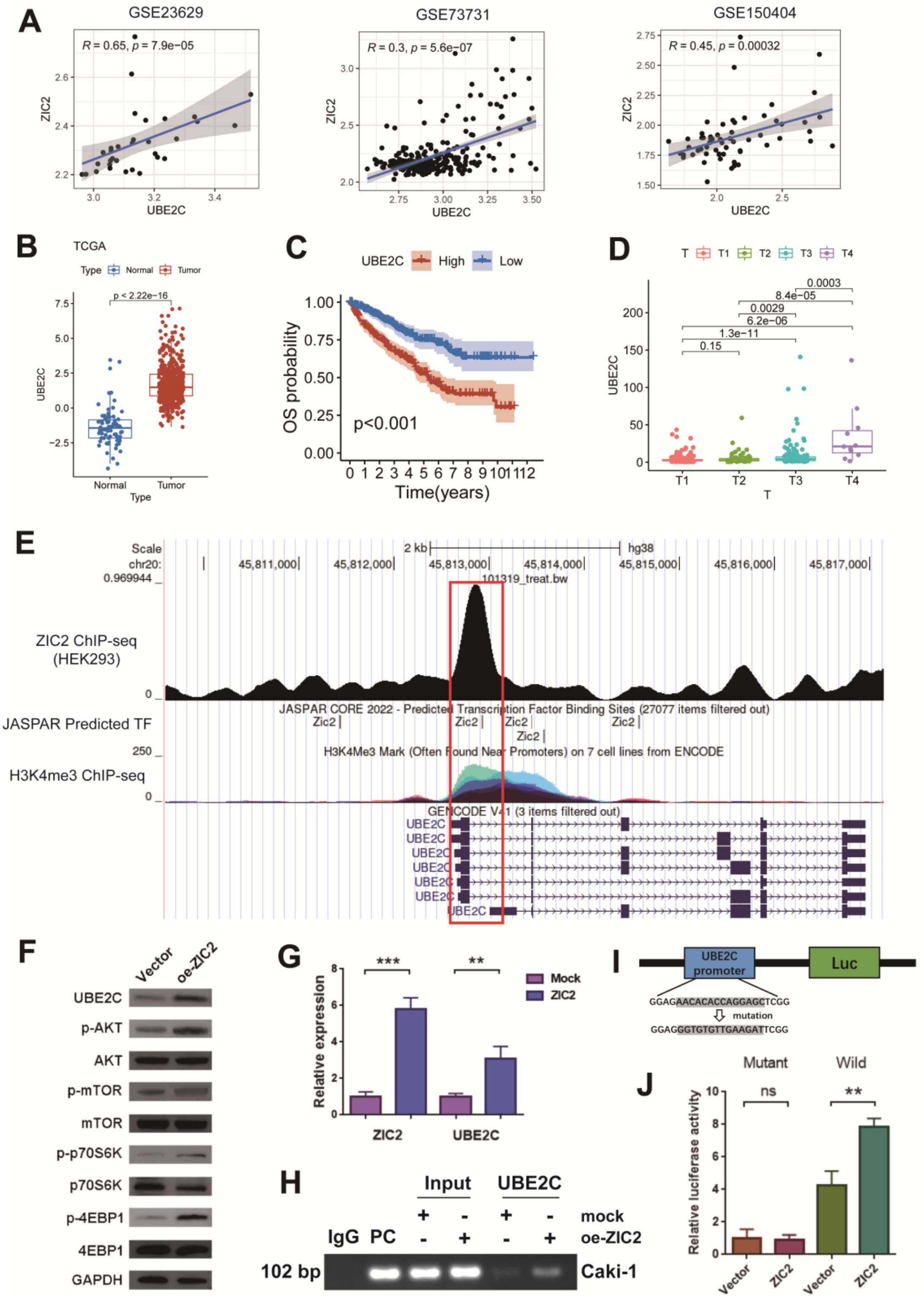

Figure S6: ZIC2 up-regulates UBE2C expression and activates AKT/mTOR signaling pathway. **(A)** UBE2C and ZIC2 expression were positively correlated in multiple data sets. **(B)** UBE2C was also highly expressed in ccRCC compared with normal tissues in TCGA. **(C)** High expression of UBE2C was associated with worse OS. **(D)** The

expression of UBE2C increased with the increase of ccRCC stage. **(E)** Chip-seq targeting ZIC2 revealed a significant sequencing peak around the UBE2C transcription start site. **(F)** Overexpression of ZIC2 increases UBE2C expression and promotes AKT/mTOR signaling. **(G)** Overexpression of ZIC2 leads to increased transcription of UBE2C. **(H)** Chip-PCR showed that overexpression of ZIC2 caused an increase in the amplification products to UBE2C promoter DNA sequence. **(I)** Construction of luciferase reporter system (wild type and mutant type). **(J)** Overexpression of ZIC2 significantly increased the luciferase activity of the wild-type luciferase reporter.
